# Supplementary material for: A combined HMM–PCNN model in the contourlet domain for image data compression
Source: PLoS One. 2020 Aug 13;15(8):e0236089. doi: 10.1371/journal.pone.0236089 (PMC7425949; doi:10.1371/journal.pone.0236089)
Supplement: Attachment — Submitted filename: response to the reviewers.pdf [file pone.0236089.s004.pdf]

Original Manuscript ID: PONE-D-19-32659

Original Article Title: **“**A combined HMM–PCNN model in the contourlet domain for image data compression ”

To: Plos One Editor

Re: Response to reviewers

Dear Editor,

There is a resubmission of our manuscript that include a response to the reviewers’ comments.
We are uploading (a) our point-by-point response to the comments (below) (response to reviewers), (b) an updated manuscript with yellow highlighting indicating changes, and (c) a clean updated manuscript without highlights (PDF main document).

Best regards,

Guoan Yang, Ph.D
Xi’an Jiaotong University

Response to the Reviewer#1

Question 1: The technical writing quality is good, although a few specific typos and corrections are given below. At a high level, the paper is not very focused, with a long rambling introduction that describes techniques at a very general level. The actual results and new techniques are really constrained to section 4, which is just 3 pages out of the 16 pages of written text.

Response 1: We would like to thank the reviewer.
We have changed the whole structure of this paper. Besides, in order to make our paper more focused, we adjusted the whole structure of the paper and implemented more experiments, more discussion is also added in the experiment part. Now, we have 21 pages in total and there is almost half of the content is about our algorithm and experiments. Also, the introduction section has been completely rewritten, and the references are changed for 33 papers (fer. 1-31, ref. 42-43). In the experiments part of the revised manuscript we have added the Table1 1, Table1 3, Table 4, Figure 14, Figure 15.

The struture of the whole paper is reorganized as follows:

1.Introduction: in this section we have rewritten the whole contents, and discussed the research progress of image coding and image compression based on multi-scale geometric analysis. Eventually, we put forward the idea to apply the HMM-PCNN model in the contourlet domain for an image data compression.
2.Theoretical preparation: in this section we introduced the contourlet transform and HMM-contourlet domain as the theoretical preparation.

3.PCNN model： in this section the parametric model of PCNN was illustrated, and then an adaptive PCNN model is presented for image data compression.

4.SPIHT algorithm: in this section we introduced the SPIHT algorithm for contourlet coding.

5.HMM/PCNN-contourlet coding using SPIHT: in this section we proposed an image data compression approach based on HMM/PCNN-contourlet coding using SPIHT algorithm. In this part, we first explained how to transfer the SPIHT algorithm from wavelet domain to contourlet domain. Then we proposed the idea to combine HMM-PCNN model with contourlet SPIHT algorithm.

6.Experiment results and analysis: in this section we selected the parameters of PCNN model and the entire experimental flowchart, eventually experiment results and analysis are given.

7.Conclusion: in this section the conclusion and further research are discussed.

Question 2: The one item I believe must be corrected is the use of the "Lena" (or "Lenna") image. While this image was used a lot in the past, the image has a long and controversial history, having originated as a scan of a Playboy centerfold. While the copyright issues have largely been resolved (primarily by the copyright holder looking the other way), the use of this image has been strongly discouraged for the past 5 years or more. There are plenty of public images that can be used that don't have the same history of sexism, and I believe Lena should not be used at all in current papers.

Response 2: We would like to thank the reviewer.
We have removed all image and experiment results related to the test image Lena.

Question 3(1): Abstract: Change "achieved a better performance" to "achieved better performance"
Response 3(1): in abstract, we change "achieved a better performance" to "leads to better compression performance and offer a more flexible encoding scheme."

Question 3(2): Page 1, line 2: Change "used by humans" to "used by most humans"
Response 3(2): "used by humans" is deleted.

Question 3(3): Page 2, lines 63-66: The authors mention work at three different universities and say they "achieved great success," but no references are given. At least some references should be included here to support the statement and provide a way for readers to look up the referenced work.
Response 3(3): this paragraph is deleted.

Question 3(4): Page 3, definitions of terms (between lines 103 and 104): It looks like text was put in math mode in latex (based on the poor formatting of the word "offspring" and "coefficient". This should be put in text mode so that the formatting is correct.
Response 3(4): in this paragraph, we put in text mode between lines 143 and 146 in the revised manuscript.

Question 3(5): Page 4, lines 114-115 (first two lines of "Initialization" pseudocode): The order of these two lines should be swapped (compute N0 before using it!).
Response 3(5): the order of these two lines is swapped between lines 158 and 159 in the revised manuscript. And we unified "N0"as "N"

Question 3(6): Page 4, line 117: What is "Phi" representing here? I would guess that it's the empty set -- if that's correct, it should be typeset in Latex using \emptyset.
Response 3(6): we change "Phi" to " typeset in Latex using \emptyset" in line 161 in the revised manuscript.

Question 3(7): Page 5, line 137: The variable "N" here was called "N_0" above. Change one or both to be consistent.
Response 3(7): the variable "N" and "N_0" are unified as "N" in line 178-180 in the revised manuscript.

Question 3(8): Page 8, Figure 6: I'm almost certain that these are labeled incorrectly in the caption. The caption says (a) is the original image, but (c) is clearly the highest quality image so should be the original. The authors need to double-check this and make sure the right image is in the right place.
Response 3(8): there are labeled incorrectly in the caption. We corrected the caption in the revised manuscript, as follows:

"Fig 9. SPIHT based on a contourlet with 4,4,8,8 directions at each level; (a) Original image, (b) Reconstructed image after coding with 0.15 bpp, (c) Reconstructed image after coding with 0.3 bpp. It can be observed that (c) is clearer than (b)"

Question 3(9): Page 8, line 215: "these coefficient" should be "these coefficients"

Response 3(9): we change "these coefficient" to "these coefficients" in line 85 page 3 in the revised manuscript.

Question 3(10): Page 16, line 482: "0.15 bpp" is not a "compression ratio" -- it's a bit rate. This is again called a "ratio" in table headings, and should be corrected to "rate" in all places where "bpp" is used.

Response 3(10): we change "compression ratio" to "compression rates" in line 342 page 15 in the revised manuscript.

We would like to thank the reviewer. The all the mistakes mentioned we have corrected.

Response to the reviewer#2

Question 1: Comparison of proposed method with other state of the art image compression algorithms is needed.

Response 1: We would like to thank the reviewer.

We have added the comparisons with standard SPIHT algorithm and a recent method named set partitioning system coding scheme, which was published in IEEE Transactions on Image Processing in 2016, i.e. reference number 48 in the revised manuscript..

Actually we did some comparisons with other state of the art image compression algorithms at first. However, the average performance is not as good as the state of the art ones for contourlet is redundant, which makes it naturally performs not so well in terms of image compression. However, under the condition of low bit rate, some good experimental results are achieved in this paper.

It is purpose in this paper that move the hybrid HMM-ANN model in the contourlet domain for image denoising in our previous work to the image compression field. To implement an image data compression task, in our coding strategy we set the coefficients into two groups to achieve a more flexible encoding scheme. With the help of probability matrix, we use PCNN to implement the classification for subband coefficients, because PCNN possesses the two-level property.

Question 2: Authors need to be very careful while writing abbreviations and equations. Abbreviations needs to be introduced before using it e.g. SPIHT on page 3 at line no. 80 and line no. 84

Response 2: We would like to thank the reviewer.

In the resubmission, we have introduced all the abbreviations before using them. For example, some uncommon abbreviations are as follows: SPIHT is short for Set Partitioning in Hierarchical Trees; PCNN is short for Pulse Coupled Neural Network; HMM is short for hidden Markov model; EM is

short for Expectation Maximum; HVS is short for human vision system; CT is short for contourlet transform; WT is short for wavelet transform.

Question 3: Variables are not defined in the equations e.g. What is c_(i,j) n in equation 5?
c(i,j) indicates the coordinate of a certain coefficient in decomposition subband., .

Response 3: We would like to thank the reviewer.

In the resubmission, c(i,j) indicates the coordinate of a certain coefficient in decomposition subband, where i indicates the horizontal position while j indicates the vertical position, in equation 8 line 150-151 page 6 in the revised manuscript.

Question 4: Classic SPIHT algorithm on page 4 needs to be properly written with all variables defined.

Response 4: We would like to thank the reviewer.

We have rewritten the SPIHT algorithm in lines 143-146, 158-160, and 178-180 page 6-7 in the revised manuscript.

Question 3: According to ref [13] PCNN parameter setting is very important but this has not been covered in this manuscript.

Response 3: We would like to thank the reviewer.

PCNN has many parameters, it is a complicated task to ensure all the parameters are at a proper value to achieve good performance. Therefore, in the paper we use an adaptive PCNN for the parameters will adjust according to the data. Some researchers specially discuss the parameters setting problem in their work, which is not the key point in our work. In our work, once the PCNN classifies the probability matrix, its job is done. Note that there reference 13 is not cited in the revised manuscript. The reference 13 in the previous version of manuscript is as follows:

[13]. Chen, Y., Park, S., Ma, Y., Ala, R. A New Automatic Parameter Setting Method of a Simplified PCNN for Image Segmentation. IEEE Transactions on neural networks. 2011, 22(6): 880-892.

Question 4: Proper caption of figure 6 and good explanation in the text is needed.

Response 4: We would like to thank the reviewer.

As the other reviewer suggests, the alignment of the images is wrong. The image encoded with 0.15bpp is more blur than the one encoded with 0.3bpp. Now we have corrected this mistake, in lines 278-279 page 11 in the revised manuscript.

Question 5: Original image was expected in the Figure 7 and also caption and text related to Figure 7 is confusing.

Response 5: We would like to thank the reviewer.

Now we have added the original image of Barbara. Here, we offer a comparison with Eslami's work [reference 9 in the revised manuscript]. In Eslami's work, contourlet transform is improved by replacing the Laplcian Pyramid with wavelet transform. Therefore, he also uses SPIHT algorithm to verify the effectiveness. We meant to given an intuitive comparison between the contourlet transform based SPIHT and improved contourlet based SPIHT.

The text related to Figure 7 [Figure 10 in the revised manuscript] is rewritten as follows:

Fig 10. Improved contourlet-based SPIHT and contourlet-based SPIHT perfomance.(a) shows the original image. (b) shows the method in [9] , which adopts the contourlet that replaces the LP decomposition with wavelet. (c) shows the image coded with an contourlet based SPIHT. From the images, it can be observed that textures in (b) are more clear than the textures in (c)

Question 6: What is ratio of the big and ratio of the small in the table 2?

Response 6: We would like to thank the reviewer.

Because the coefficient are classified into two groups: big ones and small ones, in our coding scheme, the two parts can be coded separately. The SPIHT algorithm can be implemented on the two groups in parallel. Therefore, different coding rate can be used according to the transmission demand. Now we given explanation of the meaning of the rate in Table 2 in the revised version of manuscript, as follows:

Table 2. Image compression results of SPIHT based on the contourlet-HMM{PCNN model on different bit rate (bpp)

Groups of big coefficients; Groups of small coefficients
