# Supplementary material for: A combined HMM–PCNN model in the contourlet domain for image data compression
Source: PLoS One. 2020 Aug 13;15(8):e0236089. doi: 10.1371/journal.pone.0236089 (PMC7425949; doi:10.1371/journal.pone.0236089)
Supplement: Attachment — Submitted filename: Response to the reviewers.pdf [file pone.0236089.s005.pdf]

Original Manuscript ID: PONE-D-19-32659

Original Article Title: **“**A combined HMM–PCNN model in the contourlet domain for image data compression ”

To: Plos One Editor

Re: Response to reviewers

Dear Editor,

There is a resubmission of our manuscript that include a response to the reviewers’ comments.
We are uploading (a) our point-by-point response to the comments (below) (response to reviewers), (b) an updated manuscript with yellow highlighting indicating changes, and (c) a clean updated manuscript without highlights (PDF main document).

Best regards,

Guoan Yang, Ph.D
Xi’an Jiaotong University

Response to the Reviewers

Reviewer #1:

Question 1: The motivation of the proposed combined HMM–PCNN model should be described more clearly and consistently, and it should be reflected in the method.

Response 1: We would like to thank the reviewer.

In [41] Po and Do proposed a contourlet-HMM method that there are excellent performances for image denoising and retrieval. We know further that hybrid HMM/ANN model has been successfully applied in the field of speech recognition. That is because the combined model takes advantage of the pattern classification ability of ANN and the modeling ability of HMM in spatiotemporal use. Therefore, we would try to apply a hybrid HMM/ANN model in the contourlet domain for image processing applications such as image compression. As the third generation of ANN, PCNN model simulates the activity of neurons in cat's visual cortex that not only has good biological characteristics, does not need any training and learning, but also is easy to combine with other methods. Simultaneously PCNN model also indicates the excellent performance in the field of image fusion application. Therefore, in this paper the PCNN model is selected as an ANN model, eventually we propose a hybrid HMM/PCNN model in the contourlet domain for image compression. In brief, The HMM/PCNN model make full use of the HMM's advantages of learning ability, decoding ability, ability to process the time sequence signals, and PCNN's static classification ability.

In the version of the revised manuscript, "5.1 the method" is rewritten, as follows:

5.1 The method

In [41] Po and Do proposed a contourlet-HMM method that there are excellent performances for image denoising and retrieval. We know further that hybrid HMM/ANN model has been successfully applied in the field of speech recognition. That is because the combined model takes advantage of the pattern classification ability of ANN and the modeling ability of HMM in spatiotemporal use. Therefore, we would try to apply a hybrid HMM/ANN model in the contourlet domain for image processing applications such as image compression. As the third generation of ANN, PCNN model simulates the activity of neurons in cat's visual cortex that not only has good biological characteristics, does not need any training and learning, but also is easy to combine with other methods. Simultaneously PCNN model also indicates the excellent performance in the field of image fusion application. Therefore, in this paper the PCNN model is selected as an ANN model, eventually we propose a hybrid HMM/PCNN model in the contourlet domain for image compression. In brief, The HMM/PCNN model make full use of the HMM's advantages of learning ability, decoding ability, ability to process the time sequence signals, and PCNN's static classification ability. There are several ways to combine the HMM and a PCNN:

(1) Normalize the time sequence signal using the HMM, and then input the processed signal into the PCNN to do the classification.

(2) Use PCNN to calculate the observation matrix of HMM.

(3) Use PCNN to implement the three algorithms (forward algorithm, backward algorithm, Viterbi algorithm) in the HMM.

(4) Establish an HMM network.

Therefore, based on all the contents mentioned before, we would like to combine HMM and PCNN to optimize the Po and Do's model, then apply this model to SPIHT to validate its effectiveness.

According to Po and Do's work, the distribution of the contourlet coefficients has the following characteristics [41]:

(1) The coefficients in the same subband are distributed as a zero-mean Gaussian mixture conditioned on their parent coefficients.

(2) There are two types of coefficients within the Gaussian mixture distribution: the large ones possess a large variance and a low peak, while the small ones possess a small variance and a high peak. The former presents the edges and the latter presents the plane areas.

Based on the distribution characteristics, Po and Do successfully established an HMM contourlet domain, where the state transition matrix and state probability matrix were calculated and used. In order to combine the HMM and PCNN, we need to add PCNN to the HMM in contourlet. Inspired by the first kind of combination of HMM and PCNN mentioned above, we hope to find some features in the contourlet coefficients for PCNN to process, and the feature space found is the state probability.

The reasons for choosing state probability as classification features are as follows:

(1) the magnitudes of the coefficients are unreliable for judging coefficients being large or small, since the magnitude of a certain coefficient may be quite small, but it exists in a Gaussian distribution with large variance;

(2) Some coefficients may have near or equal probabilities of being large or small, which may lead to uncertainty in the processing results, because these coefficients cannot be well identified;

(3) the EM algorithm for the HMM when describing the same image can result in different parameters, where once the difference between the new parameter and the last parameter is less than the given threshold, the iterative process stops, which causes the process to converge to different parameter sets, leading to the uncertainty of the process result. Therefore, in order to improve the processing efficiency, we use PCNN to produce a Boolean output: to transfer the uncertainty of some coefficients' probabilities into certainty.

Therefore, to implement the classification of the state probability matrix, we adopt the PCNN mentioned above. The state probability matrix has the same multilayer and multidirectional structure as the contourlet subbands. For a certain coefficient $c_{(i,j,k),x}$(denoting the x-th coefficient of the kth subband in the jth direction at the ith scale) and its corresponding two-state probabilities $s^{l}_{(i,j,k),x}$ and $s^{s}_{(i,j,k),x}$(denoting the large state and small state, respectively), they satisfy

$$p(s^{l}_{(i,j,k),x}|c_{(i,j,k),x}) + p(s^{s}_{(i,j,k),x}|c_{(i,j,k),x}) = 1 \tag{9}$$

The state probabilities distributed in each subband are considered as "pixels" whose gray levels vary between 0 and 1. If the probabilities in the PCNN are inputted with given parameters, the PCNN produces a two-value output, where 0 represents a small state and 1 represents a large state. With this output, a clearer subband of the state probability matrix can be obtained using the Zelda test image, as shown in Fig. 6

To sum up, we first decompose the image into contourlet coefficients, then we establish an HMM in contouret domain and obtain the state transition matrix and state

probability matrix. After that, we adopt PCNN to process the state probability matrix to separate the coefficients in the same subband into two groups. Finally, we encode the coefficients with SPIHT.

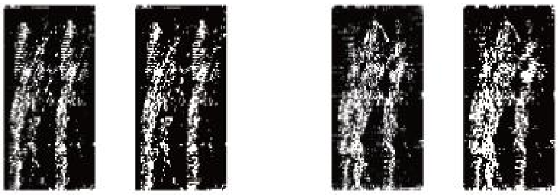

(a) (b)

Fig 6. Two subbands of the state probability of "Zelda," the leftside of each shows unprocessed subband and the rightside shows processed subband. It can be observed that the state probability processed by PCNN is clearer than the unprocessed probability and easier to classify.

Since the classification operation of coefficients is considered in our coding scheme, there is need to adjust the original coding method, just as the two classified coefficients are encoded with different compression rates and transmitted separately. After decoding, the two types of classified coefficients are recombined for reconstruction.

Question 2: There are too many experimental results in Tables 1 and Table 2, and it looks a bit confusing to analyze the experiment results. Maybe authors can adjust the table organization or bold the important results.

Response 2: We would like to thank the reviewer.

Table 1 shows the comparisons between contourlet SPIHT and wavlet SPIHT. It looks confusing indeed, and we have made the important results bold, and now it is as follow:

Table 1. Image compression performance of SPIHT

image	Butterfly				Barbara			
Rate(BPP)	0.1500	0.2000	0.2500	0.3000	0.1500	0.2000	0.2500	0.3000
WT PSNR(dB)	**27.645**	**28.203**	**28.543**	**30.125**	**24.563**	**24.998**	**25.296**	**25.677**
CT PSNR(dB)	25.947	26.80	27.130	27.296	23.023	23.585	23.843	24.010
WT SSIM	**0.7064**	**0.7592**	**0.8064**	**0.8130**	**0.6531**	**0.6730**	**0.7150**	**0.7624**
CT SSIM	0.6911	0.7178	0.7408	0.7456	0.6004	0.6329	0.6359	0.6581
image	Zelda				Goldhill			
Rate(BPP)	0.1500	0.2000	0.2500	0.3000	0.1500	0.2000	0.2500	0.3000
WT PSNR(dB)	**30.962**	**31.586**	**32.792**	**33.112**	**27.684**	**28.174**	**28.746**	**30.543**
CT PSNR(dB)	30.867	30.907	32.021	32.337	25.827	26.340	26.866	27.242
WT SSIM	**0.8306**	**0.8522**	**0.8726**	**0.8591**	**0.7138**	**0.7372**	**0.7862**	**0.8111**
CT SSIM	0.8239	0.8247	0.8524	0.8591	0.6109	0.6472	0.6750	0.6944
image	Man				Mandrill			
Rate(BPP)	0.1500	0.2000	0.2500	0.3000	0.1500	0.2000	0.2500	0.3000
WT PSNR(dB)	**24.201**	**24.784**	**25.231**	**26.464**	**20.982**	**21.203**	**21.771**	**22.364**
CT PSNR(dB)	24.014	24.606	24.932	25.169	20.690	21.194	21.438	21.777
WT SSIM	**0.6002**	**0.6529**	**0.6812**	**0.7061**	**0.4197**	**0.4884**	**0.5103**	**0.5384**
CT SSIM	0.5859	0.6238	0.6516	0.6718	0.4066	0.4632	0.4951	0.5188
image	Peppers				Camera			
Rate(BPP)	0.1500	0.2000	0.2500	0.3000	0.1500	0.2000	0.2500	0.3000
WT PSNR(dB)	**27.541**	**28.203**	**29.461**	**30.521**	**27.743**	**28.641**	**29.354**	**31.231**
CT PSNR(dB)	27.101	27.861	28.261	28.488	27.001	27.730	28.519	28.867
WT SSIM	**0.7539**	**0.7847**	**0.7948**	**0.8411**	**0.7923**	**0.8179**	**0.8388**	**0.8515**
CT SSIM	0.7354	0.7594	0.7703	0.7816	0.7653	0.7995	0.8236	0.8398

Table2 shows the performance of HMM-PCNN contourlet SPIHT, and there is no comparison. Therefore, we mainly made the PSNR and SSIM values bold, and now it is as follow:

Table 2. Image compression results of SPIHT based on the contourlet-HMM–PCNN model on different bit rate (bpp)

Image	Groups of big coefficients	Group of small coefficients	PSNR(dB)	SSIM
Butterfly	0.1500	0.1500	**26.5959**	**0.7231**
	0.2000	0.2000	**26.9279**	**0.7369**
	0.2500	0.2500	**27.0572**	**0.7474**
	0.3000	0.3000	**27.5864**	**0.7658**
Barbara	0.1500	0.1500	**23.5534**	**0.6374**
	0.2000	0.2000	**23.9111**	**0.6699**
	0.2500	0.2500	**24.2305**	**0.6867**
	0.3000	0.3000	**24.6280**	**0.7132**
Zelda	0.1500	0.1500	**31.4631**	**0.8386**
	0.2000	0.2000	**32.0071**	**0.8506**
	0.2500	0.2500	**32.0854**	**0.8667**
	0.3000	0.3000	**32.4087**	**0.8639**
Goldhill	0.1500	0.1500	**26.3568**	**0.6462**
	0.2000	0.2000	**26.9466**	**0.6778**
	0.2500	0.2500	**26.2877**	**0.7034**
	0.3000	0.3000	**27.4705**	**0.7196**
Man	0.1500	0.1500	**24.5138**	**0.6298**
	0.2000	0.2000	**24.9985**	**0.6628**
	0.2500	0.2500	**25.5123**	**0.6900**
	0.3000	0.3000	**25.7233**	**0.7097**
Mandrill	0.1500	0.1500	**21.1476**	**0.4572**
	0.2000	0.2000	**21.5482**	**0.5013**
	0.2500	0.2500	**21.8008**	**0.5303**
	0.3000	0.3000	**22.0296**	**0.5618**
Peppers	0.1500	0.1500	**27.7252**	**0.7569**
	0.2000	0.2000	**28.1414**	**0.7732**
	0.2500	0.2500	**28.4153**	**0.7872**
	0.3000	0.3000	**28.7465**	**0.7920**
Camera	0.1500	0.1500	**27.6717**	**0.7998**
	0.2000	0.2000	**28.1938**	**0.8283**
	0.2500	0.2500	**28.6629**	**0.8472**
	0.3000	0.3000	**29.1115**	**0.8628**

Question 3: The experiments should enrich the comparison between the proposed method and other state-of-the-art algorithms further. Their method is just compared with one method presented in 2016. The reviewer hopes to see more discussions and experiments to demonstrate the efficiency and robustness of the proposed method.

Response 3: We would like to thank the reviewer.

As mentioned, our method focuses on the proving the effectiveness of HMM-PCNN in contourlet, and SPIHT is adopted to reflect the effectiveness. Therefore, in the original manuscript, we mainly compare HMM/PCNN-contourlet SPIHT with contourlet SPIHT and wavelet SPIHT. And now, we find the other state of the art methods [reference 49-52] and BPG (Better portable graphics)

algorithm [51] to make more comparisons. We tested the common performance on Kodak and make comparisons with the state of the art methods, and the RD-curves and discussions are as follows:

We also compared the proposed method with several recently proposed learning based mthods including the method by Theis [49], Balle [50], the fully convolutional vector quantization network(VQNet) [51], the soft-to-hard VQ based method (SHVQ) [52], and a state-of-the art BPG method was also compared [51]. Both the PSNR and the SSIM were used to evaluate the performance of the test methods, and the test images are from Kodak. The test results are shown in Fig. 15, Fig. 16. Note that the test image Butterfly is from reference [53].

We mainly tested the algorithms with low bit rate since contourlet transform is redundant and performs poorly at high bit rate encoding. According to the PSNR results, it can be observed that at lower bit rate our model performs similarly with the methods of Theis and Balle. With the bit rate becoming larger, our method is inferior to the other methods. Generally, the BD-rate increases by 6.1% when compared with VQ-Net, reduces by 0.4% when compared with Theis's method, increases by 5.9% when compared with Balle's method. According to the SSIM result, it can be observed that our method performs not so satisfactory when compared to the other methods.

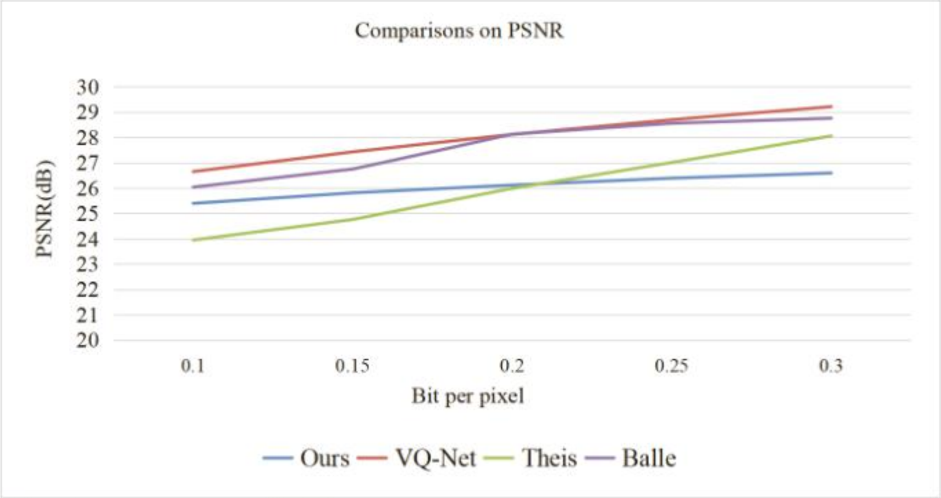

Fig 15. Comparisons on PSNR.

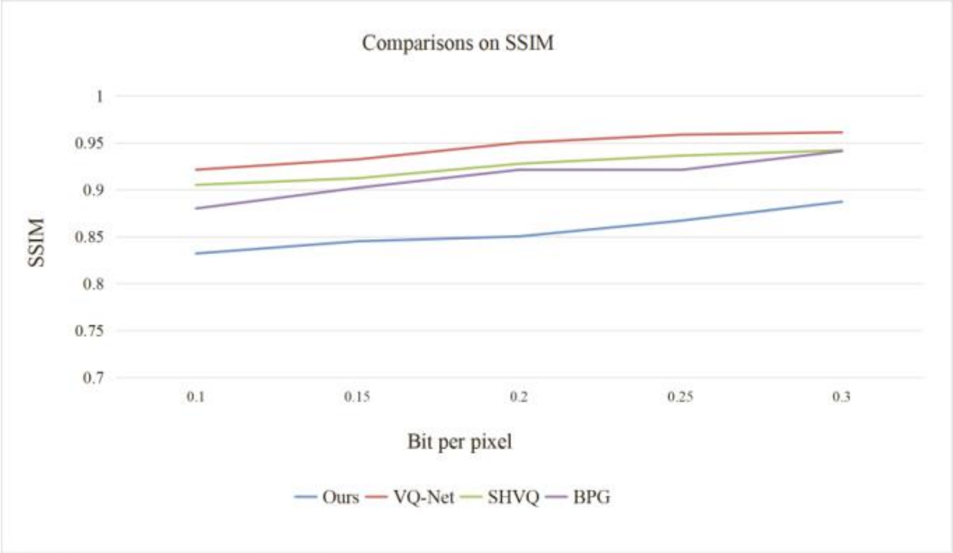

Fig 16. Comparisons on SSIM.

References

[51] Xiaotong Lu, Heng Wang, Weisheng Dong, FangFang Wu, Zhonglong Zheng, and Guanming Shi, “Learning a Deep Vector Quantization Network for Image Compression,” *IEEE Access*, vol. 7, pp. 118815-118825, 2019.

[52] E. Agustsson, F. Mentzer, M. Tschannen, L. Cavigelli, R. Timofte, L. Benini, and L. Van Gool, ‘‘Soft-to-hard vector quantization for end to-end learning compressible representations,’’ in *Proc. Adv. Neural Inf. Process. Syst.*, pp. 1141–1151, 2017.

[50] J. Ballé, V. Laparra, and E. P. Simoncelli, ‘‘End-to-end optimized image compression,’’ International Conference on Learning Representations, ICLR2017, Toulon, France, April 24-26, 2017, vol. 1, pp. 1-27.

[49] L. Theis, W. Shi, A. Cunningham, and F. Huszár, ‘‘Lossy image compression with compressive autoencoders,’’ International Conference on Learning Representations, ICLR2017, Toulon, France, April 24-26, 2017, vol. 1, pp. 1-19.

[53] Jianzhong Li, An optimized watermarking scheme using an encrypted gyrator transform computer generated hologram based on particle swarm optimization, Optics Express, 2014, vol. 22, no. 8, pp. 10001-10016.

Reviewer #2

Question 1: The author should spend more descriptions on the method they proposed. It is confused to introduce too many methods used before. In the meanwhile, although the author focus on a different transform for image compression, there is still lack of comparison with SOTA learned compression methods. More intuitive data should be provided such as BD-rate reduction based on a specific anchor.

Response 1: We would like to thank the reviewer.

As mentioned, our method focuses on the proving the effectiveness of HMM-PCNN in contourlet, and SPIHT is adopted to reflect the effectiveness. Therefore, in the original manuscript, we mainly compare HMM/PCNN-contourlet SPIHT with contourlet SPIHT and wavelet SPIHT. And now, we find the other state of the art methods [reference 49-52] and BPG (Better port graphics) algorithm to make more comparisons. We tested the common performance on Kodak and make comparisons with the state of the art methods, and the RD curves instead of BD-rate and discussions are as follows:

We mainly tested the algorithms with low bit rate for performance comparison. Because the contourlet transform is redundant and thus performs poorly at high bit rate encoding. According to the PSNR results, it can be observed that at lower bit rate our model performs similarly with the methods of Theis and Ballé. With the bit rate becoming larger, our method is inferior to the other state of the art methods. Generally, the BD-rate increases by 6.1% when compared with VQ-Net, reduces by 0.4% when compared with Theis's method, increases by 5.9% when compared with Ballé's method. According to the SSIM result, it can be observed that our method performs not so satisfactory when compared to the other state of the art methods.

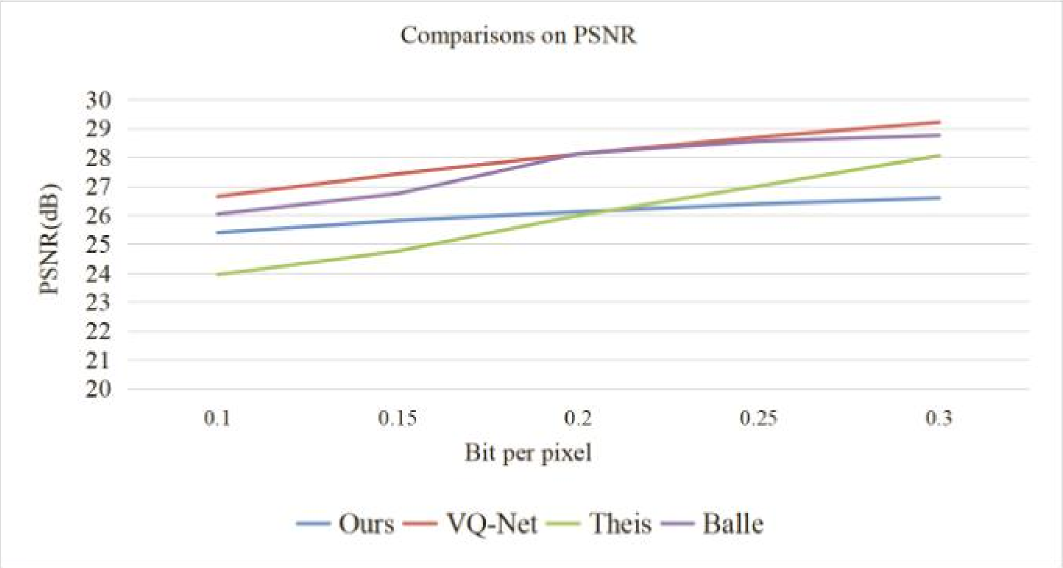

Fig 15. Comparisons on PSNR.

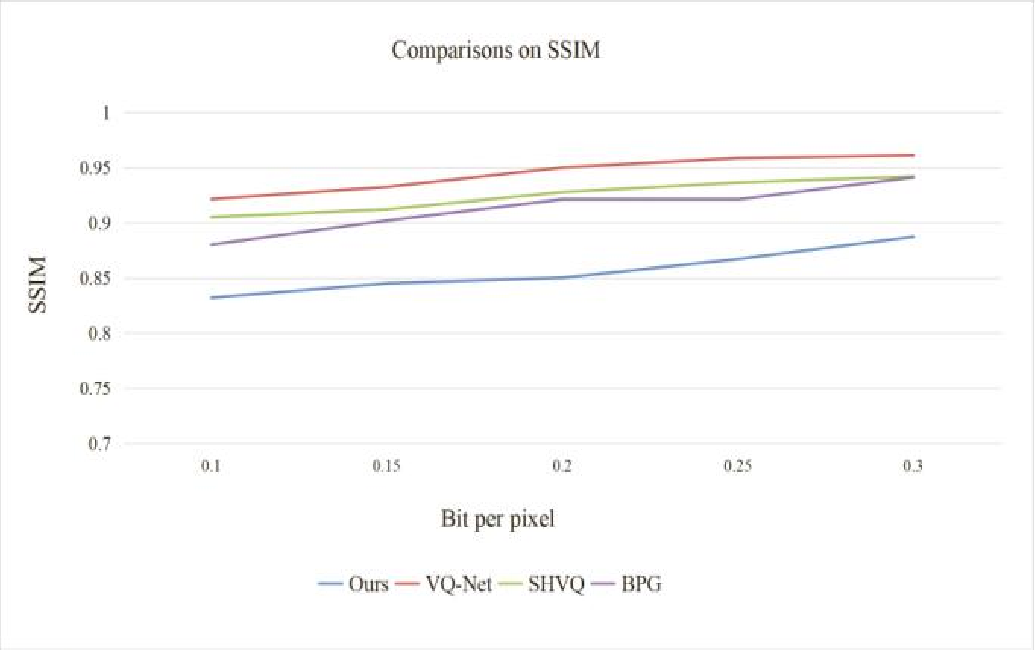

Fig 16. Comparisons on SSIM.

References
[51] Xiaotong Lu, Heng Wang, Weisheng Dong, FangFang Wu, Zhonglong Zheng, and Guanming Shi, "Learning a Deep Vector Quantization Network for Image Compression," *IEEE Access*, vol. 7, pp. 118815-118825, 2019.
[52] E. Agustsson, F. Mentzer, M. Tschannen, L. Cavigelli, R. Timofte, L. Benini, and L. Van Gool, ''Soft-to-hard vector quantization for endto-end learning compressible representations,'' in Proc. Adv. Neural Inf. Process. Syst., 2017, vol. 1, pp. 1141–1151 .

[50] J. Ballé, V. Laparra, and E. P. Simoncelli, ''End-to-end optimized image compression,'' International Conference on Learning Representations, ICLR2017, Toulon, France, April 24-26, 2017, vol. 1, pp. 1-27.

[49] L. Theis, W. Shi, A. Cunningham, and F. Huszár, ''Lossy image compression with compressive autoencoders,'' International Conference on Learning Representations, ICLR2017, Toulon, France, April 24-26, 2017, vol. 1, pp. 1-19.

[53] Jianzhong Li, An optimized watermarking scheme using an encrypted gyrator transform computer generated hologram based on particle swarm optimization, Optics Express, 2014, vol. 22, no. 8, pp. 10001-10016.

Question 2: Common test results on well-known data Kodak should be provided.

Response 2: We would like to thank the reviewer.

In the response to question 1, we responded that we've obtained the common results on Kodak.
